# BSEMS—A Blockchain-Based Smart Energy Measurement System

**DOI:** 10.3390/s23198086

**Published:** 2023-09-26

**Authors:** Manmeet Singh, Suhaib Ahmed, Sparsh Sharma, Saurabh Singh, Byungun Yoon

**Affiliations:** 1 Department of Information Technology and Engineering, Baba Ghulam Shah Badshah University, Rajouri 185234, India; mannirulz@gmail.com; 2 Department of Computer Science and Engineering, Chitkara University Institute of Engineering and Technology, Chitkara University, Punjab 140401, India; sabatt@outlook.com; 3 Computer Science & Engineering, National Institute of Technology Srinagar, Jammu and Kashmir 190006, India; sparsharma@outlook.com; 4 AI and Big Data, Woosong University, Daejeon 34606, Republic of Korea; singh.saurabh@wsu.ac.kr; 5 Department of Industrial & Systems Engineering, Dongguk University, Seoul 04620, Republic of Korea

**Keywords:** blockchain, energy monitoring, energy management, smart energy measurement

## Abstract

The modern world’s increasing reliance on automated systems for everyday tasks has resulted in a corresponding rise in power consumption. The demand is further augmented by increased sales of electric vehicles, smart cities, smart transportation, etc. This growing dependence underscores the critical necessity for a robust smart energy measurement and management system to ensure a continuous and efficient power supply. However, implementing such a system presents a set of challenges, particularly concerning the transparency, security, and trustworthiness of data storage and retrieval. Blockchain technology offers an innovative solution in the form of a distributed ledger, which guarantees secure and transparent transaction storage and retrieval. This research introduces a blockchain-based system, utilising Hyperledger Fabric and smart contracts, designed for the secure storage and retrieval of consumers’ energy consumption data. Finally, a user-friendly web portal was designed and developed using the node.js framework, offering an accessible and intuitive interface to monitor and manage energy consumption effectively.

## 1. Introduction

Global energy consumption and production continue to rise steadily [1]. Over the past decade, there has been a notable transformation in energy production, primarily driven by automation, scientific advancement, and the rapid growth of leading nations worldwide. The increasing reliance of humanity on automated systems for everyday tasks is also contributing to a daily surge in power usage. Moreover, the increasing demand for electric vehicles is also contributing to the global market demand [2]. The rising demand necessitates a fitting reaction through the implementation of a smart energy management and monitoring system.

Smart management and control strategies must be used to reduce required investment, but these jobs are becoming more difficult as energy systems become more dynamic, decentralised, complex, and “multiagent”, with a greater number of actors and potential outcomes. A growing amount of the power network’s components demand sophisticated communication and data exchanges, which increases the difficulty of central control and operation. To support these decentralisation and digitalisation tendencies, local distributed control and management approaches are needed [3]. The main purpose of blockchains, also known as distributed ledger technologies (DLT), is to make distributed transactions easier by doing away with central management.

Blockchains are distributed data structures or ledgers that allow for the secure storage of digital transactions without the need for a central authority. More crucially, blockchains enable peer-to-peer (P2P) networks to automatically execute smart contracts [4,5,6,7]. Blockchains can alternately be considered databases that let several users update the ledger at once, thereby creating various chain versions. Each member of the network has a copy of the records chain, and all members use a consensus to agree on the ledger’s valid state rather than having a single trusted centre manage it.

The following are the main contributions of this paper.

1. Analysing and comparing different energy measurement systems based on blockchain technologies.
2. Proposing an architecture for a blockchain-based smart energy measurement system.
3. Developing and testing a blockchain-based smart energy measurement system called BSEMS using smart meters and Hyperledger technology.
4. Creating a test web portal, built using the node.js framework, for user interaction with BSEMS.

The subsequent sections of this paper are structured as follows: Section 2 delves into a comprehensive literature review, encompassing an introduction to blockchain, an exploration of how blockchain operates, an analysis of the features inherent to blockchain technology, and a discussion of potential applications of blockchain in smart energy measurement systems, followed by an examination of Hyperledger Fabric. Section 3 subsequently presents related work in the domain of blockchain-based smart energy measurement systems. Section 4 provides an in-depth examination of the proposed system’s architecture, followed by an elucidation of its key components. Section 5 is dedicated to presenting the results obtained. Finally, Section 6 encapsulates the paper’s conclusion.

## 2. Literature Review

Blockchain technology has recently attracted much interest in handling several issues related to data management and security. It could find applications in various sectors including the energy sector [8], where it can drastically change the monitoring and consumption of energy resources.

The application of blockchain to smart energy measurement can provide a transparent, decentralised, and trustworthy platform for the effective monitoring and management of energy consumption. Blockchain-based smart electric meters should be resistant to any kind of energy-related fraud and manipulation, thereby building trust among consumers.

### 2.1. Introduction to Blockchain

Blockchain is a distributed and decentralised ledger that is used to store and manage digital transactions [9]. Its decentralised nature ensures that data are not controlled by a single centralised entity; instead, every node participating in the blockchain network possesses a copy of the entire blockchain.

In 2008, Satoshi Nakamoto introduced the first blockchain application: Bitcoin P2P electronic cash. Bitcoin is completely based on blockchain technology, and it allows users to perform transactions without requiring centralised authorities like banks.

Subsequently, researchers have explored the potential of blockchain technology to enhance trust and transparency in various other domain areas like supply chains, smart contracts, and Industry 4.0 [10].

### 2.2. Working of Blockchain

**Figure 1:**
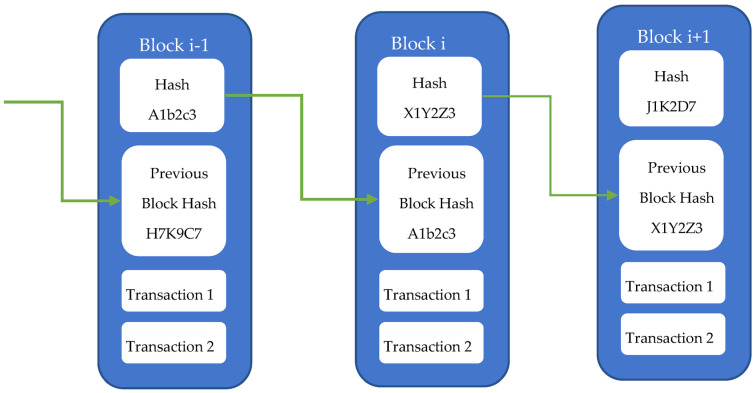
Blockchain structure.

Blockchain technology involves the following steps:

1. Transaction Verification: First, transactions are verified. Every time some node wants to initiate any transaction, that transaction is sent out to every node in the blockchain network for verification. Upon receiving the verification request, all nodes examine whether the transaction is legitimate and whether it is suitable for insertion in the blockchain network.
2. Block Creation: Once the transaction is verified, a block is created, and the transaction is added to it. Each block has a header, transactions, and a hash of itself. Figure 1 presents the structure of the blockchain wherein each block usually consists of multiple verified transactions. Further, blocks are connected to each other through the special previous hash field in their header; this field contains the hash value of the previous block.
3. Consensus: Consensus ensures that the block is added to the blockchain network only if all nodes in the network agree that it is genuine. Toward this end, a consensus mechanism like Proof of Work is used.
4. Verification and Mining: Nodes must check whether a new block satisfies the requirements for inclusion when it is added to the chain. Mining is the process of validating blocks by completing challenging mathematical puzzles. Cryptocurrency rewards are given to nodes who successfully validate a block, thus encouraging them to participate in the network.

### 2.3. Features of Blockchain Technology

Blockchain provides various opportunities but also involves challenges [11]. The key features of blockchain technology are as follows:

1. Decentralisation: The main characteristic of blockchain technology is that it is decentralised, in that no central authority or middleman controls the system. Instead, the network comprises numerous nodes, each of which has a copy of the ledger. This increases the system’s security and transparency because no single point of failure or corruption exists.
2. Transparency: In blockchain technology, a transaction is accessible to all nodes in the network once it is recorded on the blockchain. Therefore, everyone may observe its specifics, including its value, date and time, and participants. This openness guarantees that everyone involved understands the transaction clearly and aids in preventing fraud and deception.
3. Immutability: A transaction cannot be changed or removed after it has been added to the blockchain. This is because each block in the blockchain has its own cryptographic hash, which is produced depending on the block’s data. The block’s hash will change if any of the data are changed, making it easy to detect tampering. Blockchain technology is the best choice for applications that require secure and tamper-proof records because this immutability assures the integrity of the data.
4. Security: In blockchain technology, modern cryptographic methods are used to guarantee transaction security. A single node cannot modify or fabricate data because numerous nodes in the network validate and verify each transaction. Furthermore, the use of public and private keys guarantees that only those with the proper authorisation may access the data.
5. Smart contracts: Self-executing contracts known as “smart contracts” are built into the blockchain. They make it possible to automate some portions of a transaction, like money transfer or delivery, and may reduce the need for middlemen. To ensure that all parties understand the terms and circumstances of the contract, smart contracts are made tamper-proof and transparent.
6. Speed and efficiency: Blockchain technology can conduct transactions much more quickly and effectively than conventional systems. This is because transactions are not slowed down by middlemen or manual verification procedures. Blockchain technology is particularly suitable for applications that need high-speed and high-volume processing because it can handle a very large number of transactions concurrently.

### 2.4. Potential Applications of Blockchain in Smart Energy Measurement Systems

Blockchain applications have been discussed previously [12]. The applications of blockchain in smart energy measurement systems are as follows:

1. Energy trading: Blockchain technology facilitates P2P transactions between energy providers and consumers, thus simplifying the trade of energy. It avoids the need for middlemen and reduces transaction costs. Smart contracts automate the trading process and ensure that all parties adhere to the conditions of the agreement of the transaction.
2. Grid management: Blockchain can perform grid management more effectively. The ability to monitor energy use in real time can enable grid managers to balance supply and demand. Grid operators may use blockchain to guarantee the confidentiality and safety of data collected through smart meters.
3. Renewable energy certificates: Renewable energy certificates (RECs) may be produced and exchanged using blockchain technology. They serve as evidence that a specific amount of electricity was produced using renewable energy sources. Energy producers may be adequately rewarded for what they contribute to the grid by trading RECs in a transparent and secure manner by using blockchain.
4. Energy storage: Blockchain may also be used to manage and improve energy storage systems by ensuring that energy is retained in reserve and distributed when it is most needed. This may reduce waste and increase the total efficiency of the energy system.
5. Energy efficiency: Blockchain can be used to incentivise energy efficiency by creating a transparent and secure platform for rewarding consumers who reduce their energy consumption. Further, it can be used to efficiently and inexpensively implement energy efficiency programs.

### 2.5. Hyperledger Fabric

**Figure 2:**
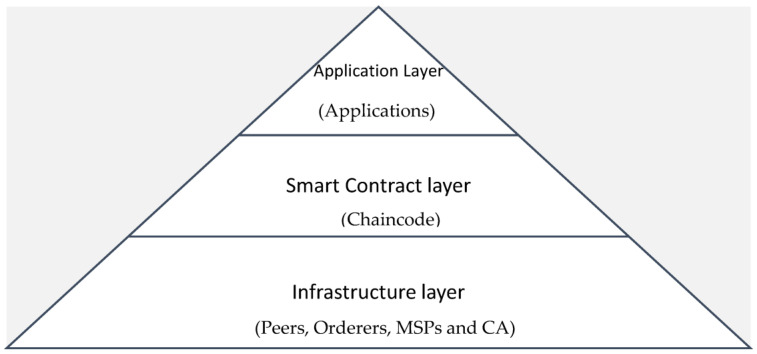
Hyperledger Fabric application stack.

The Linux Foundation hosts the Hyperledger project [13] and is responsible for the development of the Hyperledger Fabric distributed database. It was intended to be flexible, scalable, and safe and has become one of the most popular enterprise blockchain systems. The open-source Hyperledger Fabric framework enables businesses to create their own decentralised blockchain networks and manage them according to their needs. The architecture of Hyperledger Fabric can be divided into three main layers as shown in Figure 2.

1. Application Layer: The application layer provides an interface for third-party applications to interact with the Hyperledger Fabric network. This layer includes client software and peer nodes. Client apps may communicate with peer nodes to transmit transaction requests; peer nodes will then execute these transactions and update the ledger.
2. Smart Contract Layer: The smart contract layer, also called the chaincode layer, implements the network’s business logic [14]. Hyperledger Fabric supports programming languages such as Go, Java, and Node.js for the creation of smart contracts. The ledger transactions can be performed by chaincodes distributed via a variety of channels.
3. Infrastructure Layer: The infrastructure layer comprises various elements of the network’s underpinning infrastructure, including ordering nodes, peer nodes, and certificate authorities.

A comprehensive examination of the utilisation of blockchain technologies within the realm of smart grids is presented in [15]. Incorporating blockchain into smart grids offers multiple advantages. The study delves deeply into the integration of blockchain in home automation, EV charging, and advanced metering infrastructure. Moreover, the research places a strong emphasis on addressing security and privacy vulnerabilities in blockchain-based energy management systems. Additionally, it explores various countermeasures against these potential attacks.

As 5G adoption becomes more widespread, it is anticipated that the Industrial Internet of Things (IIoT) will experience substantial growth, particularly in sectors such as manufacturing, smart transportation [16], energy, and smart cities [17]. This accelerated growth is expected to generate vast amounts of data, giving rise to concerns regarding data privacy. Cao et al. [18] have introduced an upgraded algorithm aimed at enhancing the scalability and decentralisation of private blockchains while simultaneously reducing latency and cost.

## 3. Related Work

**Figure 3:**
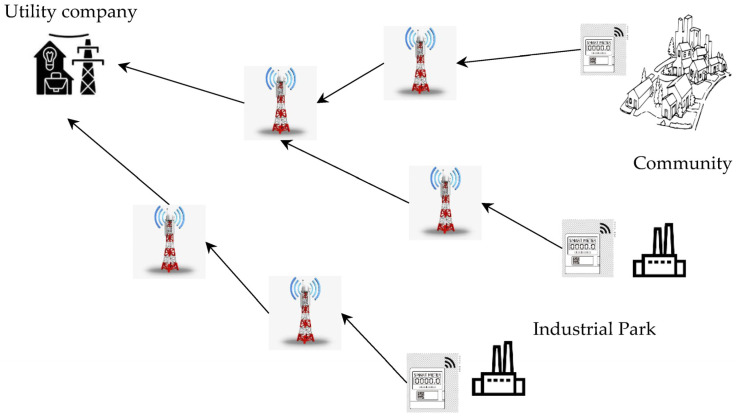
System architecture for electricity anomaly detection proposed in [20].

One study [19] used blockchain technologies in energy monitoring and measurement for the detection of abnormalities in electricity use in industrial wireless sensor networks. Another study [20] focused on the difficulties posed by the growing volume of big data produced by Industry 4.0 and the potential repercussions of improper and delayed anomaly detection. This study evaluated existing anomaly detection techniques and proposed an anomaly detection mechanism that uses distributed sensor processing, smart meter readings, machine learning, and big data analysis to accurately and effectively identify anomalies in power use. Figure 3 shows the proposed model. This model uses an Industrial Internet of Things framework built on a blockchain that supports distributed and cooperative anomaly detection. This mechanism involves data collection, data training, anomaly detection, and anomaly precaution stages. In keeping with the locations of anomaly reporting sensors, anomalies are found, and possible hazards are mined to stop mishaps. To find abnormalities that may appear normal based on their findings, data mining methods like k-nearest neighbour are used. This technique can successfully detect anomalies in both SGs and industrial sensor networks.

**Figure 4:**
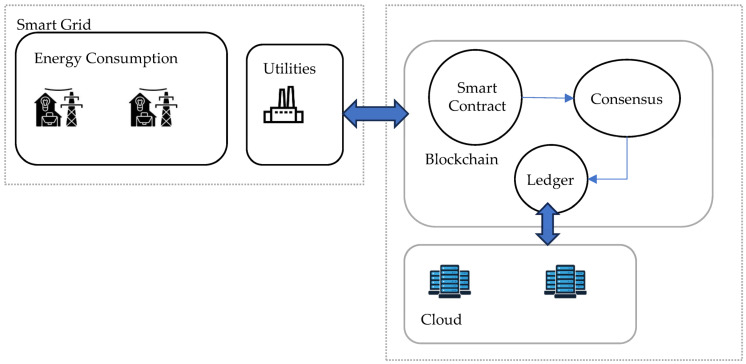
System model proposed in [21].

Better energy distribution is required to meet the current demands of these vast groupings of electronic devices. Energy companies can use SGs to more easily distribute electricity to customers in accordance with their unique needs. Blockchain is a promising technology that offers the functionality needed to address the majority of problems in this area, such as preserving large amounts of data, deleting data, tampering with data, and revising data. Additionally, it avoids the need for middlemen. Owing to its distributed structure and inherent security, it is the ideal choice for enhancing services as a whole. Smart contracts have been improved such that the price per unit is dynamically determined depending on renewable energy resources and utility-generated energy units in the overall grid [21]. Additionally, the system is automated so that electricity is moved from one resident (or service) to another in accordance with their needs. A smart contract is used to exchange energy after determining each participant’s needs. At the time of registration, each participant specifies their prerequisites and can subsequently change these. Figure 4 shows the proposed system model. The two primary contributions of this paper are (1) using smart contracts to automate the bidding process for energy-related transactions based on supply and demand and (2) combining blockchain and Hyperledger Fabric and Composer so that consumers can enjoy dynamic pricing based on supply while maintaining privacy, anonymity, and secrecy.

Aiman et al. [22] used Wattcoin to create a P2P token-based billing system for energy provision. They implemented smart contracts and kept track of transactions using the Ethereum virtual engine. Smart meters were used to keep track of energy use, and an exhaustion contract was used to update the Wattcoin wallet’s token information. The revised token was then sent to the energy provider’s account.

Zhang et al. [23] proposed a double auction system based on consortium blockchain to reduce costs and increase productivity while protecting the anonymity of transactions. Both buyers and sellers must submit bids on the energy price to obtain competitive costs for energy use. At both the supplier and customer levels, energy costs are properly sorted based on the bid values. The supplier offers energy to the customer based on the properly matched value.

Jindal et al. [24] proposed a safe demand response system based on blockchain technology called GUARDIAN for addressing challenges in energy trading for domestic, commercial, and industrial demands. There are two different types of nodes in the system: miner nodes and regular nodes. The miner node’s responsibility is to oversee all energy-related SG transactions, including authentication and authorisation. Normal nodes often have smaller ledger sizes than those of mining nodes.

Abu-Amara et al. [25] proposed a blockchain-based solution for tracking electricity and water consumption, viewing bills, and securely completing bill payments in Abu Dhabi. This solution provided traceability, transparency, and consistency and reduced costs in the business network. Users of this solution use the BlockchainWE Mobile App, and the blockchain hashes and maintains the login and registration information received by the application.

Waseem et al. [26] introduced a scheduling system for household appliances that utilises Grey Wolf and Crow Search Optimisation (GWCSO) to address the challenges of optimising energy demand and generation. Their approach, known as the Innovative Home Appliance Scheduling (IHAS) framework, aims to reduce costs, improve user comfort, and analyse the peak-to-average ratio. They conducted a case study specifically focused on scheduling air conditioners to assess the algorithm’s efficiency. The results demonstrate a significant improvement in cost reduction and peak-to-average ratio. It is worth noting that this study concentrated on household loads, which are less complex and demanding compared to industrial loads.

Blockchain technology is being leveraged to address issues and tackle the complexities associated with energy consumption and generation in both residential and industrial settings. Some research efforts are dedicated to detecting anomalies in energy consumption to identify instances of energy theft, while others are focused on addressing the challenges related to energy demand and response by utilising advanced algorithms such as Grey Wolf and Crow Search Optimisation. There is a growing demand for research into the application of blockchain in smart energy management systems, particularly within campus environments. This study aims to develop a blockchain-based smart energy management system that facilitates interaction through a web portal.

## 4. Technique

In this section, we describe the overall architecture and hardware devices and software used for the proposed blockchain-based smart energy measurement system (BSEMS).

### 4.1. Architecture

**Figure 5:**
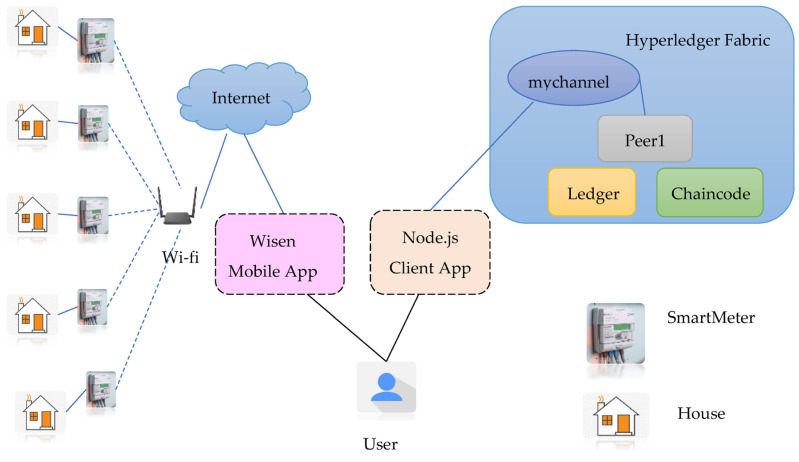
BSEMS architecture.

Figure 5 presents the detailed architecture of the BSEMS. It consists of smart Wi-Fi meters, a Wisen mobile app, a node.js client application, and a Hyperledger Fabric network. The Wisen mobile application [27] was used to record the energy consumption data from the smart Wi-Fi meters at a given point in time. The recorded values can then be submitted to the blockchain network by using the node.js client application.

### 4.2. Components

This section describes the various hardware and software used along with a detailed description of the various functions used in the smart contract.

#### 4.2.1. Smart Wi-Fi Meters

**Figure 6:**
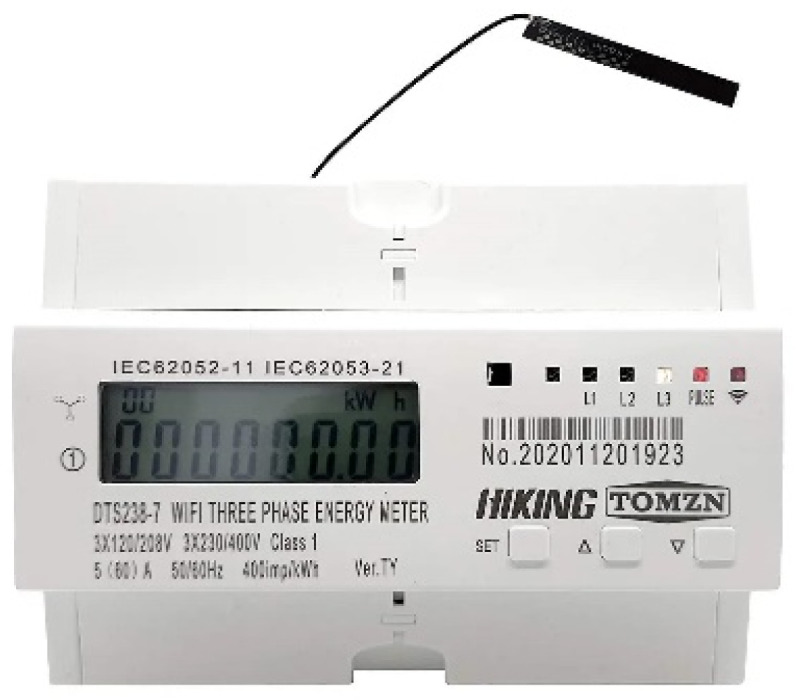
Smart Wi-Fi meter.

This study used three-phase smart Wi-Fi meters from AmiciSmart [28]. These smart meters can be configured and installed easily by following the setup guide. Figure 6 shows the smart meter used in the system. This meter provides various configuration settings for under/over voltage and overload protection.

#### 4.2.2. Wisen Mobile Application

**Figure 7:**
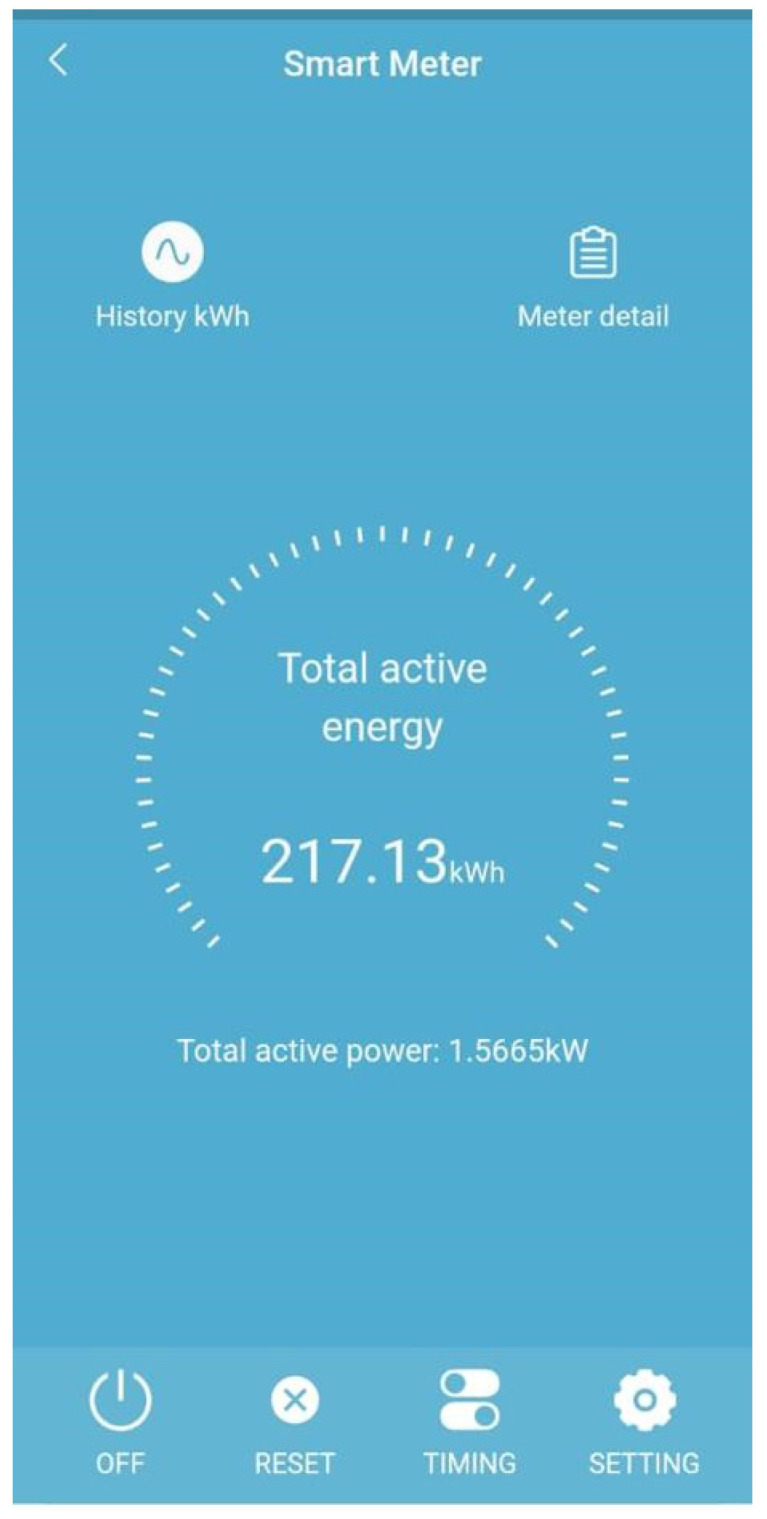
Wisen App meter reading.

Wisen [27] is a mobile application for controlling smart devices at home. It can connect to smart Wi-Fi meters at home and obtain meter readings at a given point in time; it can also turn the meters on/off remotely. Figure 7 shows a screenshot of a meter reading on the Wisen mobile application.

#### 4.2.3. Hyperledger Fabric

Hyperledger Fabric [13] is an open-source DLT developed for enterprise applications. It is a permissioned blockchain technology that allows transactions from registered members only. It allows the use of smart contracts (chaincode) to interact with the distributed ledger by using channels while offering both privacy and confidentiality.

#### 4.2.4. IBM Blockchain Platform

IBM blockchain platform [29] is a blockchain-as-a-service provided by IBM to build and deploy applications by using a blockchain network. IBM blockchain platform extension for vs. code [30] can be used freely to build and deploy smart contracts on the Hyperledger Fabric network. Furthermore, applications can be deployed by using the network.

#### 4.2.5. Smart Contract

A smart contract [14] consists of a set of rules to interact with the distributed ledger via transactions. Applications can invoke a smart contract to interact with the distributed ledger to, for example, create a new transaction or read the world state, as shown in Table 1. In the smart contract, various transactions are available to manage customer-related data. The “myCustomerExists” function serves as a read-only check to determine whether a customer ID already exists within the system. To create a new customer with a specific customer ID, the “createMyCustomer” transaction is used. To retrieve energy consumption data for a particular customer, the “readMyCustomer” function is employed, while the “updateMyCustomer” function allows for the modification of energy consumption data for a specified customer. When it becomes necessary to remove customer entries from the system, the “deleteMyCustomer” function is utilised. Lastly, the “queryAllCustomer” function provides a read-only option to access energy consumption data for all customers within the system. These transactions collectively facilitate the management of customer-related information in the system.

## 5. Discussion and Results

**Figure 8:**
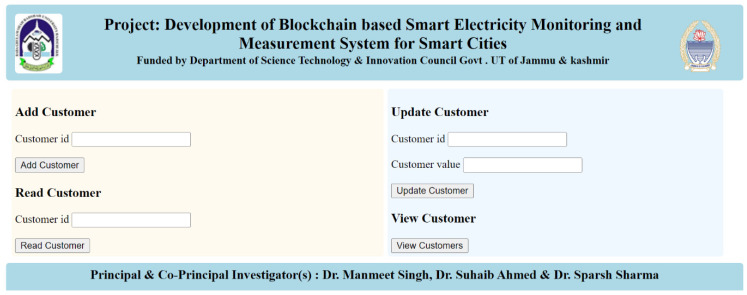
Website homepage.

In this section, we will delve into the comprehensive implementation of the proposed system, offering a detailed overview of its functionality and user interface. The system’s access point is the homepage, which is visually depicted in Figure 8, providing users with a user-friendly and intuitive starting point for engaging with the system’s capabilities.

Upon landing on the homepage, users are presented with a diverse range of options, each tailored to accommodate specific interactions with the system. Among these options are the following key functionalities:

Adding a New Customer: Users have the ability to seamlessly integrate new customers into the system. By selecting this option, they can initiate the process of creating a customer profile, specifying a unique customer ID, and inputting relevant information.

Reading Customer Energy Data: The system offers a feature that enables users to retrieve comprehensive energy consumption data for individual customers. Through this option, users can access historical and real-time energy consumption statistics, facilitating data-driven decision-making.

Updating Customer Energy Data: To maintain accurate and up-to-date records, users can employ the “update a customer’s energy data” functionality. This option permits users to make necessary modifications to a customer’s energy consumption data, ensuring that the information remains current and reflective of actual usage patterns.

By offering these distinct but interconnected functionalities, the system empowers users to efficiently manage customer-related data and make informed decisions regarding energy consumption. This user-friendly interface simplifies the navigation process, enhancing the overall user experience and streamlining interactions with the system’s core features.

The system’s versatility in managing customer energy data is evident through two core features: the “update customer page” and the “View customers data page”. When it comes to maintaining accurate and current energy consumption records, the “update customer page” proves invaluable. By inputting the customer’s unique identifier, known as the customer ID, along with the updated energy value, users can effortlessly ensure that the system reflects the most recent data regarding energy consumption, aligning the records with real-time usage patterns.

**Figure 9:**
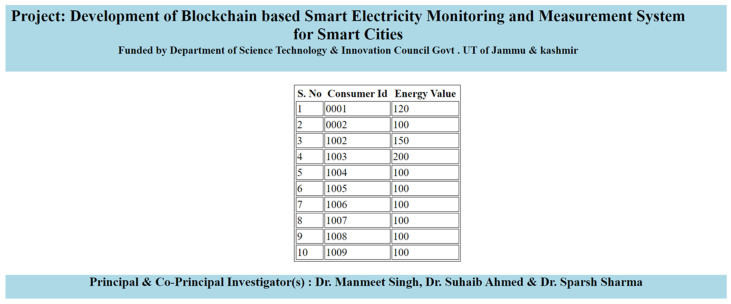
View consumers’ energy data in kilowatt-hours (kWh).

In parallel, the “View customers data page” offers an insightful and user-friendly way to access a comprehensive overview of energy data for all customers. This information is presented in a structured tabular format, as vividly depicted in Figure 9. Beyond its aesthetic appeal, this page serves as a pivotal resource for data analysis, enabling users to discern consumption trends, identify outliers, and make data-driven decisions. Moreover, this page plays a crucial role in validating the effectiveness of updates made via the “update customer page”, as it provides a holistic view of the updated energy values alongside existing data, further enhancing the system’s utility in managing and visualising customer energy information.

**Figure 10:**
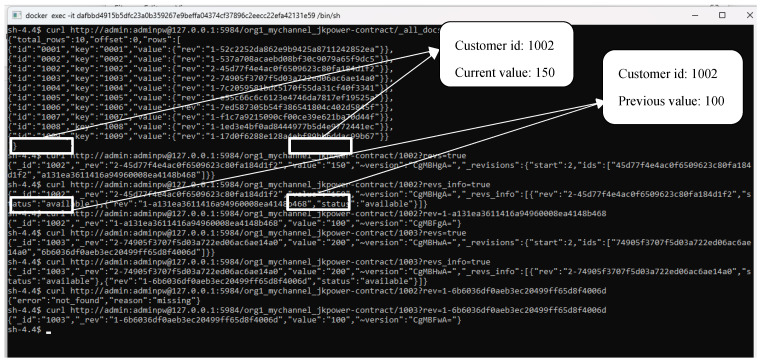
Blockchain network using CouchDB to store consumer’s energy data.

Figure 10 provides a clear visualisation of the customer data stored within the CouchDB database, which operates in conjunction with the blockchain network. Taking into account the scenario involving a customer with a customer ID of 1002, the customer’s present energy consumption stands at 150 units, specifically in kilowatt-hours (kWh), while the previous consumption was recorded at 100 units. This storage system goes beyond merely recording energy values; it also incorporates the use of hashes to bolster data integrity. These cryptographic hashes serve as a vital security measure, safeguarding against unauthorised alterations or tampering of the stored information, thus ensuring the trustworthiness of the data.

One noteworthy aspect is that the CouchDB database supports querying capabilities, enabling users to access and review historical records of customer energy data. This functionality proves invaluable for retrospective analysis, allowing users to trace the evolution of energy consumption patterns over time or verify the accuracy of past data entries. In essence, the integration of CouchDB within the blockchain network not only secures the data but also empowers users with the ability to access and retrieve historical customer energy data, fostering transparency and enhancing the system’s utility for data management and analysis.

## 6. Conclusions

In summary, this study has pursued several key objectives in the realm of energy measurement and blockchain technology. Firstly, it involved a meticulous analysis and comparison of various energy measurement systems that leverage blockchain technologies, aiming to identify their strengths, weaknesses, and potential for enhancing energy management practices. Subsequently, a novel architecture was proposed for a blockchain-based smart energy measurement system.

The practical implementation of these concepts led to the creation and rigorous testing of the blockchain-based smart energy measurement system (BSEMS), effectively showcasing the feasibility and efficiency of this innovative approach. By leveraging smart meters and Hyperledger technology, BSEMS has demonstrated its capacity to securely and accurately record energy consumption data, enhancing transparency and reliability in energy management.

Lastly, to facilitate user engagement and interaction with BSEMS, a user-friendly web portal was designed and developed using the node.js framework, offering an accessible and intuitive interface for individuals to monitor and manage their energy consumption effectively. Collectively, this work has not only contributed valuable insights into the integration of blockchain technology with energy measurement systems but has also yielded a practical and user-oriented solution in the form of BSEMS.

It will be interesting to see the performance of the proposed system in the real world where there are large numbers of consumers. The smart meters used in the current study did not provide API to access consumers energy data, hence the need for manual lookup and updating on the portal. In the future, human intervention can be avoided if there are Cloud API or other means to connect/query the smart meters directly at periodic intervals.

## Figures and Tables

**Table 1:** Smart contract transactions.

Transaction	Description
myCustomerExists	This read-only function checks whether a customer ID already exists or not.
createMyCustomer	Create a new customer with a given customer ID.
readMyCustomer	This read-only function returns the energy consumption data of a given customer.
updateMyCustomer	This function updates the energy consumption data of a given customer.
deleteMyCustomer	This function is used to delete entries for a given customer ID.
queryAllCustomer	This read-only function returns the energy consumption data of all the customers.

## Data Availability

No new data were created.
